# Mitochondrial DNA methylation profiling of the human prefrontal cortex and nucleus accumbens: correlations with aging and drug use

**DOI:** 10.1186/s13148-022-01300-z

**Published:** 2022-06-25

**Authors:** Chia-Hung Huang, Man-Chen Chang, Yung-Chun Lai, Chun-Yen Lin, Cho-Hsien Hsu, Bo-Yuan Tseng, Chuhsing Kate Hsiao, Tzu-Pin Lu, Sung-Liang Yu, Sung-Tsang Hsieh, Wei J. Chen

**Affiliations:** 1 Forensic Biology Division, Institute of Forensic Medicine, Ministry of Justice, New Taipei City, Taiwan; 2 Forensic Pathology Division, Institute of Forensic Medicine, Ministry of Justice, New Taipei City, Taiwan; 3 Institute of Epidemiology and Preventive Medicine, National Taiwan University, Taipei, Taiwan; 4 Department of Clinical Laboratory Sciences and Medical Biotechnology, College of Medicine, National Taiwan University, Taipei, Taiwan; 5 Department of Anatomy and Cell Biology, College of Medicine, National Taiwan University, Taipei, Taiwan; 6 Department of Neurology, College of Medicine and National Taiwan University Hospital, National Taiwan University, Taipei, Taiwan; 7 Department of Psychiatry, College of Medicine and National Taiwan University Hospital, National Taiwan University, Taipei, Taiwan; 8 Center for Neuropsychiatric Research, National Health Research Institutes, Zhunan, Miaoli County, Taiwan

**Keywords:** Epigenetic, Whole-mitochondrial-genome bisulfite sequencing, Aging, Illicit drug, Nucleus accumbens, Prefrontal cortex

## Abstract

**Background:**

Despite the brain’s high demand for energy, research on its epigenetics focuses on nuclear methylation, and much of the mitochondrial DNA methylation remains seldom investigated. With a focus on the nucleus accumbens (NAcc) and the prefrontal cortex (PFC), we aimed to identify the mitochondrial methylation signatures for (1) distinguishing the two brain areas, (2) correlating with aging, and (3) reflecting the influence of illicit drugs on the brain.

**Result:**

We collected the brain tissue in the NAcc and the PFC from the deceased individuals without (*n* = 39) and with (*n* = 14) drug use and used whole-genome bisulfite sequencing to cover cytosine sites in the mitochondrial genome. We first detected differential methylations between the NAcc and the PFC in the nonusers group (*P* = 3.89 × 10^–9^). These function-related methylation differences diminished in the drug use group due to the selective alteration in the NAcc. Then, we found the correlation between the methylation levels and the chronological ages in the nonusers group (*R*^2^ = 0.34 in the NAcc and 0.37 in the PFC). The epigenetic clocks in illicit drug users, especially in the ketamine users, were accelerated in both brain regions by comparison with the nonusers. Finally, we summarized the effect of the illicit drugs on the methylation, which could significantly differentiate the drug users from the nonusers (AUC = 0.88 in the NAcc, AUC = 0.94 in the PFC).

**Conclusion:**

The mitochondrial methylations were different between different brain areas, generally accumulated with aging, and sensitive to the effects of illicit drugs. We believed this is the first report to elucidate comprehensively the importance of mitochondrial DNA methylation in human brain.

**Supplementary Information:**

The online version contains supplementary material available at 10.1186/s13148-022-01300-z.

## Background

Mitochondria are the power generator in almost all cell types, and their function is especially critical to energy-demanding tissues such as the brain [1]. Mitochondria also interact with the nuclear genome to participate in metabolism [2] and apoptosis [3]. From an evolutionary perspective, mitochondria play a vital role in cell survival and are critical sensors in the cell that enable the cell to cope with environmental challenges [4]. Following the identification of an independent genome in the mitochondria (mtDNA), recent studies have indicated that, similar to the epigenetics of nuclear DNA (nDNA), various mechanisms of gene regulation are also involved in the epigenetics of mtDNA [5, 6].

During human brain development, maturation, and learning, dynamic nuclear DNA methylation changes have been observed [7]. A growing body of literature has indicated that certain cognitive functions or behaviors are associated with nuclear DNA methylation changes in particular brain regions, e.g., changes related to cigarette smoking in the nucleus accumbens (NAcc) [8], changes related to human immunodeficiency virus infection and methamphetamine use in the frontal cortex [9], and differential methylation among four brain regions enriched with genes related to neuropsychiatric disorders [10]. Furthermore, nuclear DNA methylation levels have been used to predict age across a broad spectrum of human tissues, including the brain [11], and this predicted epigenetic age represents a health status named the epigenetic clock [12, 13]. Heroin use might alter the nuclear epigenetic clock in the prefrontal cortex (PFC) [14]. Despite the brain being an energy-demanding organ, where neurons need to have local energy usage that is spatially matched to energy production by the mitochondria [15], much remains unknown about the methylation patterns in its mitochondria.

Major challenges in studying mitochondrial DNA methylation are its relatively low level of methylation [16, 17] and sequence similarity to the nuclear mitochondrial DNA (NUMTs) [18], rending its detection difficult [19]. Nevertheless, there have been studies that successfully measured mitochondrial DNA methylation in the blood [16] and hepatocytes [20] and reported strand-biased methylation in mitochondrial DNA [21], demonstrating applicable methods to deal with the NUMTs [22, 23]. Compared with the nuclear genome, the mitochondrial genome has been found to have higher non-CpG methylation rather than CpG methylation [24]. Interestingly, non-CpG methylation in the nucleus is involved in energy regulation [25]. To date, there has been only one study examining mitochondrial methylation in the brain [26], which methodically avoided the overestimation of mitochondrial DNA methylation but was limited to CpG methylation. Whether the mitochondrial DNA methylation pattern is associated with aging and whether it is influenced by illicit drug use remain unknown.

To fill this gap in the literature, we sequenced both CpG and non-CpG methylation of the mitochondria in the postmortem brain tissues of two groups of deceased individuals: a control group and a drug use group. With a focus on the NAcc and the PFC, which are involved in different functions, we aimed to examine (1) whether the methylation pattern of the NAcc is different from that of the PFC; (2) whether there is a correlation between aging and the methylation profile in the brain; and (3) whether illicit drug use selectively affected either function-related or age-related methylation patterns, or whether it broadly affected whole mitochondrial DNA methylation. Our results provide missing information on mitochondrial DNA methylation and pave the way to a better understanding of the epigenetics of the brain.

## Results

### Characteristics of the study samples

We collected male forensic patients of Taiwanese ethnicity to minimize the influence of sex and ethnicity in this research. Based on the toxicological reports, the deceased individuals were divided into (1) a control group (*n* = 39), consisting of deceased individuals with negative toxicological tests for heroin, amphetamine-type stimulants (ATSs), and ketamine, and (2) a drug use group (*n* = 14), consisting of deceased individuals with a toxicologically confirmed presence of any illicit drugs. The number of deceased individuals who tested positive for individual illicit drugs was 2 for heroin, 5 for ATSs only (all involving methamphetamine), 3 for ketamine only, and 4 for both ATSs (1 methamphetamine, 1 ecstasy, and 2 mephedrone) and ketamine (more details in Additional file 1: Figure S1). Most of the deceased individuals in both groups were inferred to be free from alcohol intake before death and had a postmortem interval of less than 6 h (Table 1). Reflecting a typical pattern in forensic practices, the members of the drug use group were younger and had their causes of death determined to be less due to natural death and more due to homicide compared with the control group.

**Table 1:** Characteristics of the deceased individuals in this study

	Control (*N* = 39)	Drug use^a^ (*N* = 14^b^)	Group comparison *P* value
Age (year), mean (SD)	54.9 (21.5)	37.5 (12.0)	0.006
Alcohol intake, *n* (%)	7 (18.0)	2 (14.3)	0.754
Postmortem interval ≤ 6 h, *n* (%)	32 (82.0)	9 (64.3)	0.173
Cause of death, *n* (%)			0.015
Accident	17 (43.6)	4 (28.6)	
Homicide	3 (7.7)	5 (35.7)	
Suicide	2 (5.1)	3 (21.4)	
Natural death	15 (38.5)	1 (7.1)	
Undetermined	2 (5.1)	1 (7.1)	

^a^This group included deceased individuals who had used heroin, amphetamine-type stimulants (ATSs) or ketamine

^b^One of the heroin users without adequate methylation data for the PFC

### Low mitochondrial methylation levels in both the NAcc and the PFC

In deriving our results of mitochondrial methylation, we minimized the potential influence from the NUMTs in three steps. First, we isolated mitochondria to eliminate most of the nuclei. Second, we sequenced the reads with lengths up to 300 bp (before quality check) for alignment to gain an accuracy as high as possible [27]. Lastly, we only analyzed the reads uniquely aligned to the mitochondrial genome, with any reads aligned with more than one region being deleted from this study.

**Fig. 1:**
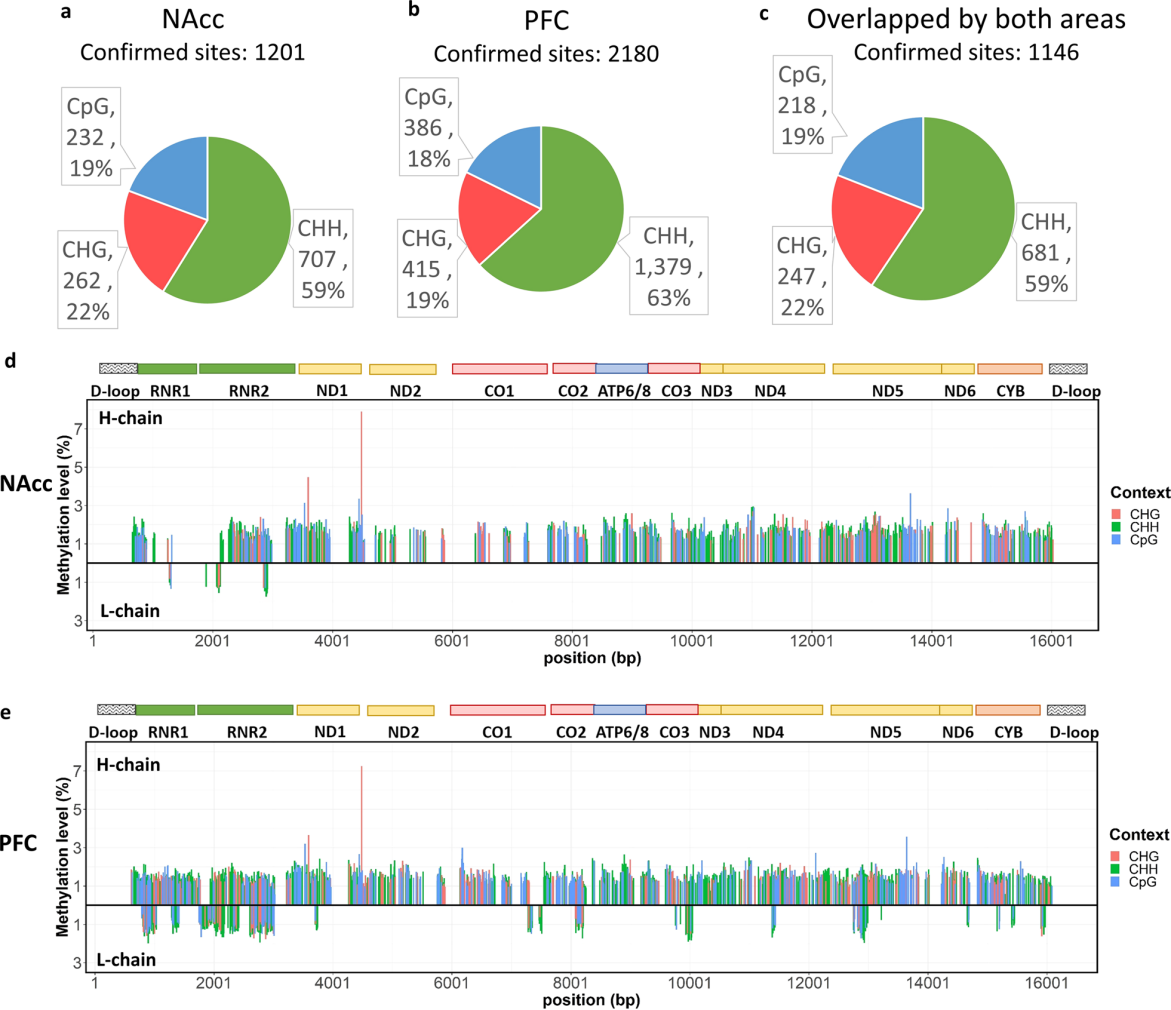
Mitochondrial methylation profiles in the NAcc and the PFC. **a**–**c** The pie charts show the confirmed cytosine sites and the sequence contexts. The context, amount and proportion of cytosine sites are labeled in the plots. **d**, **e** The bar plots show the methylation levels of the cytosine sites on the H-chain and the L-chain (as indicated) across the mitochondrial genome of the brain areas. The color of the bar represents the sequence context of cytosine, and the encoded genes are shown at the top of the plot.

Among the cytosine sites from both strands of the mitochondrial DNA using bisulfite sequencing, the methylation level of a cytosine site was confirmed if it passed quality assurance (Q-score ≥ 30, coverage ≥ 30) and was detectable in all samples. In total, we confirmed the methylation levels of 1201 cytosine sites from the NAcc and 2180 cytosine sites from the PFC, with 1146 cytosine sites overlapping between both areas. The distributions of three types of sequence contexts (i.e., CHH, CHG, and CpG; H being A, C or T) among the sequenced cytosines are provided in Fig. 1a–c. Cytosines with confirmed methylation were more frequently reported in the H-chain than in the L-chain (Fig. 1d, e). For cytosine sites without confirmed methylation, some were on the L-chain with a coverage of < 30, and some were due to differences in polymorphisms between Europeans and Asians. Based on those confirmed cytosine sites, both the CpG and non-CpG methylation levels in the NAcc were mostly approximately 2%; even among those with higher methylation levels, the highest average methylation level was <8%. Similarly, relatively low levels of methylation patterns (approximately 2%) were observed in the PFC.

### Differential methylations between the NAcc and the PFC

**Fig. 2:**
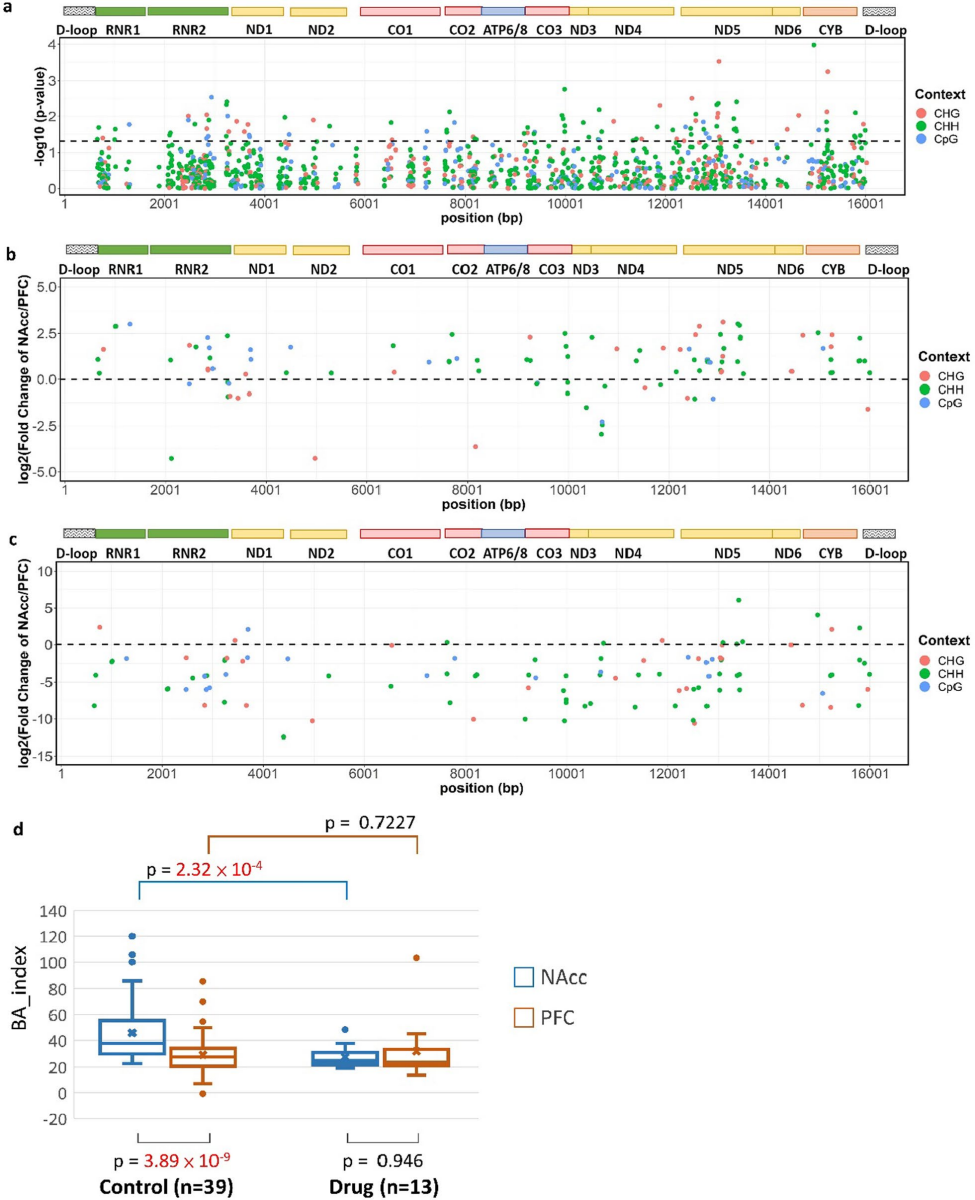
The brain area index (BA_index) in the control group and the drug use group. **a** for the differential methylation levels between the NAcc and the PFC, the Manhattan plot shows the significance level of each cytosine site across the mitochondrial genome. The cytosine sites above the dashed line (*P* value = 0.05) were selected for constructing the BA_index, and their fold changes of methylation levels between the NAcc and the PFC are shown in **b**. **c** The scatter plot shows the fold changes of methylation levels between the NAcc and the PFC from the selected cytosine sites in the drug use group. **a**–**c** The encoded genes are shown at the top of each plot, and the color of the dot represents the sequence context. **d** The box plot shows the distribution of the BA_index scores in different brain areas and groups. The *P* values are shown in the plots.

We then evaluated whether the two brain areas with different functions could have different methylation signatures in the mitochondrial genome. Among the overlapping cytosine sites (*n* = 1146) between the NAcc and the PFC in the control group (i.e., the nonusers group), their methylation levels differed in 105 sites, as determined using the Wilcoxon signed rank test for matched samples (*P* value < 0.05) (Fig. 2a). For cytosine sites with differential methylation, the contexts were not limited to CpG, and at most sites, the NAcc had higher methylation levels than the PFC (Fig. 2b). The details of those cytosine sites are listed in Additional file 2: Table S3. To summarize the differential methylation between the NAcc and the PFC in the control group, we constructed a brain area index (BA_index) by summing the methylation levels of those 105 cytosine sites using the logarithm of the fold change in each site as its weight. As expected, the BA_index scores in the NAcc were greater than those in the PFC (*P* value = 3.89 × 10^–9^, Fig. 2d) in the control group. In addition, the corresponding BA_index scores in the drug use group were not different between the NAcc and the PFC (*P* value = 0.94) (Fig. 2c). When each brain area was compared between the two groups, the drug use group had lower BA_index scores in the NAcc than the counterpart in the control group (*P* value = 2.32 × 10^–4^) but had similar BA_index scores in the PFC to the counterpart in the control group (*P* value = 0.72).

### The correlation between aging and methylation levels in the mitochondrial genome

**Fig. 3:**
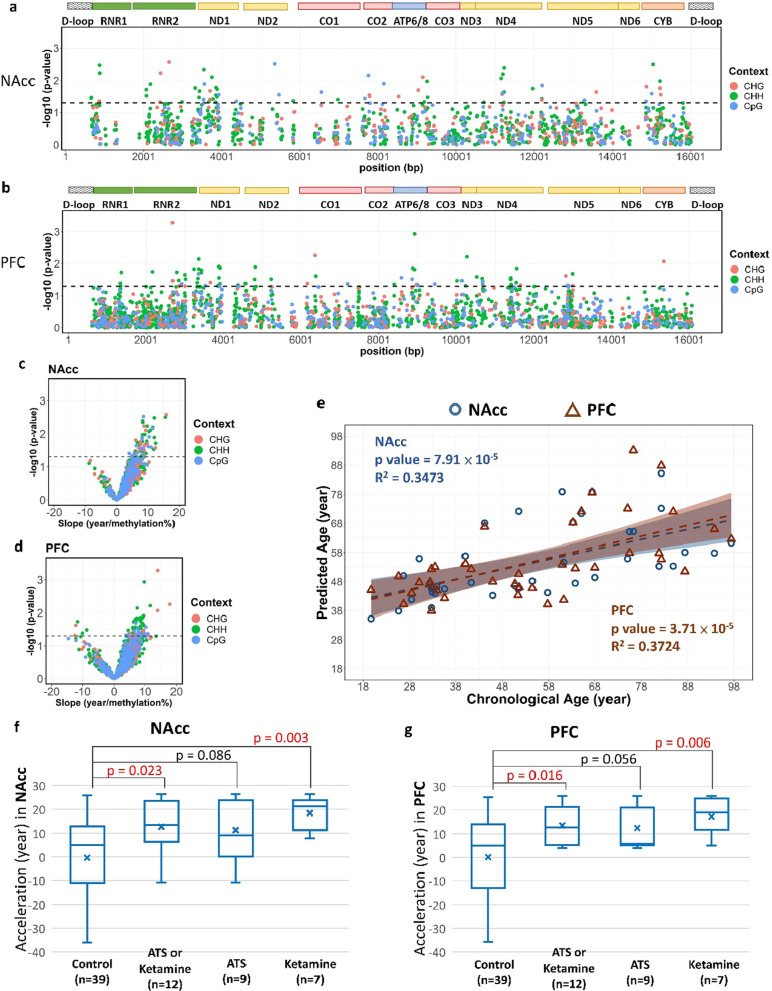
The mitochondrial epigenetic clock in the NAcc and the PFC. For the correlations between methylation levels and chronological ages, the Manhattan plots shows the significance level of each cytosine site across the mitochondrial genome of the NAcc (**a**) and the PFC (**b**). The encoded genes are shown at the top of the plots. In **c** and **d**, the volcano plots show the correlations between methylation levels and chronological ages in the NAcc (**c**) and the PFC (**d**). **a**–**d** The cytosine sites above the dashed line (*P* value = 0.05) were selected for constructing the Age_index, and the color of each dot represents the sequence context. **e** The scatter plot shows the correlations between the chronological ages and the predicted ages from the NAcc (○) and the PFC (∆). The blue shade area (NAcc) and the brown shade area (PFC) represent the 95% confidence intervals for the predicted line in each region. The *R*^2^ and *P* value are indicated in the plot. **f**, **g** The box plots show the distribution of the accelerated ages in different groups in the NAcc (**f**) and the PFC (**g**). The *P* values are shown in the plots.

Next, we examined the epigenetic clock in the mitochondrial genome. In the control (nonusers) group, both the methylation levels of 70 cytosine sites (including 15 CpG sites) in the NAcc and those of another set of 70 cytosine sites (including 13 CpG sites) in the PFC were correlated with individuals’ chronological ages (Pearson’s correlation, *P* value < 0.05), with the majority of them being positively correlated (Fig. 3a–d). The details of those cytosine sites are listed in Additional file 2: Table S6 for the NAcc and Table S7 for the PFC. We constructed an Age_index by summing the methylation levels of those cytosine sites using Pearson’s correlation coefficient (slope) at each site as its weight. When the chronological age was regressed on the Age_index in the control group, the predicted ages were highly correlated with the chronological ages in both the NAcc and the PFC (*P* value = 7.91 × 10^–5^ and 3.71 × 10^–5^, respectively, Fig. 3e), and the *R*^2^ was 0.3473 in the NAcc and 0.3724 in the PFC, with the predicted lines along their confidence intervals for the two regions highly overlapping.

Using the Age_index derived from the control group, i.e., an index of epigenetic age, we then evaluated whether there was age acceleration in the drug use group by subtracting the chronological age from the predicted age. Since there were only 2 deceased individuals who tested positive for heroin, which has been found to have a rejuvenating effect [14] rather than an age acceleration effect, these individuals were excluded from this part of the analysis. The results revealed that there was indeed an acceleration in the epigenetic age among the deceased individuals with the presence of ATSs or ketamine (*n* = 12) in both the NAcc (an increase of 8.5 years in the median; *P* value = 0.023) and the PFC (an increase of 7.7 years in the median; *P* value = 0.016) compared with the control group (Fig. 3f, g). When individuals with ATSs and individuals with ketamine in their systems were examined separately, a prominent age acceleration was found among those deceased individuals with the presence of any ketamine (*n* = 7) in both the NAcc (an increase of 16.1 years in the median; *P* value = 0.003) and the PFC (an increase of 13.9 years in the median; *P* value = 0.006), and a borderline significant age acceleration among those deceased individuals with the presence of any ATS (*n* = 9) in both the NAcc (*P* value = 0.086) and the PFC (*P* value = 0.056).

### General influence of drug use on cytosine methylation in the mitochondrial genome

**Fig. 4:**
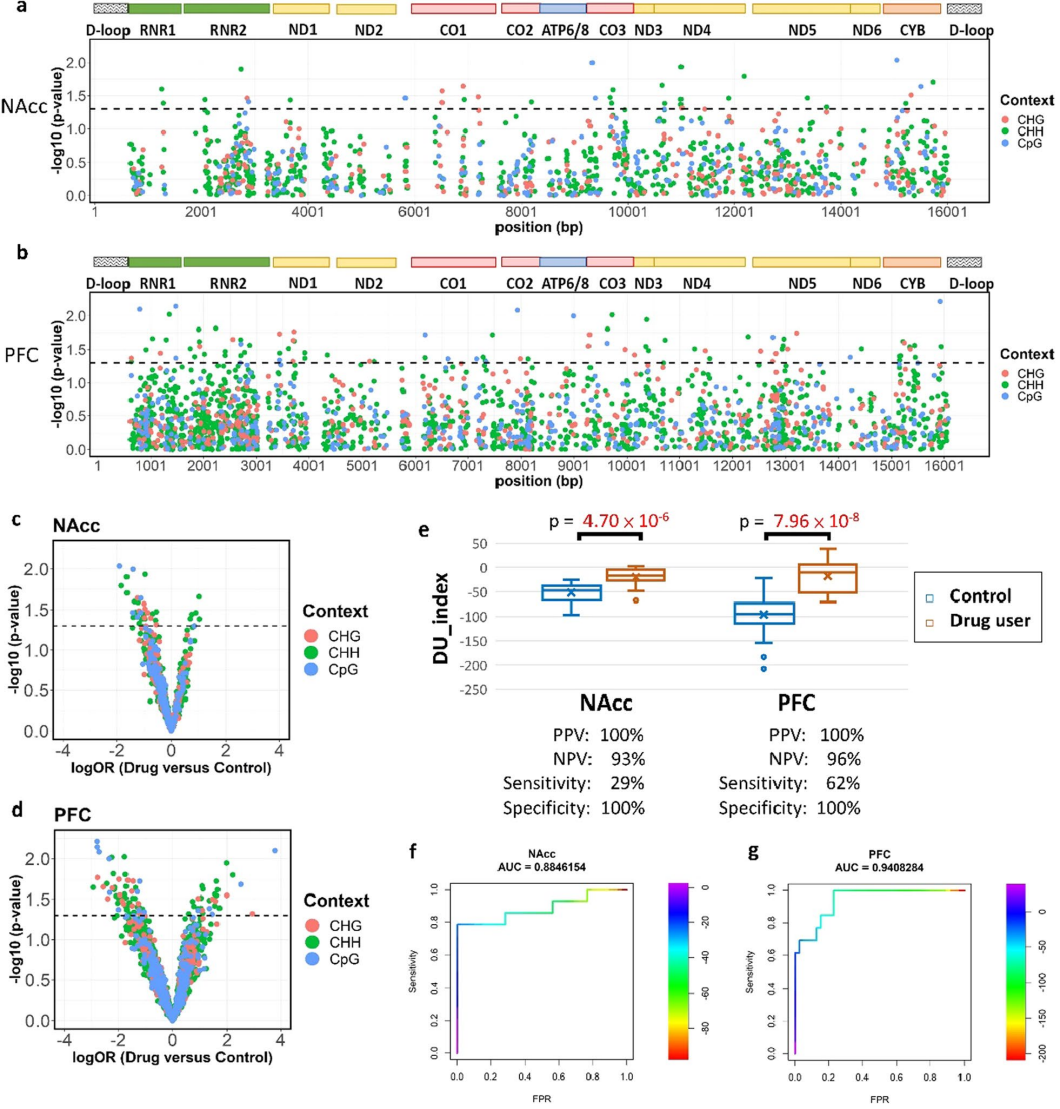
The Drug-using index (DU_index) in the NAcc and the PFC. For the differential methylation levels between the drug use group and the control group, the Manhattan plots show the significance level of each cytosine site across the mitochondrial genome of the NAcc (**a**) and the PFC (**b**). The encoded genes are shown at the top of the plots. **c**, **d** The volcano plots show the relative methylation level of each cytosine between the drug use group and the control group against the *P* value in the NAcc (**c**) and the PFC (**d**). **a**–**d** The cytosine sites above the dashed line (*P* value = 0.05) were selected for constructing the DU_index, and the color of each dot represents the sequence context. **e** The box plot shows the distribution of the DU_index scores in different groups and the brain areas. The *P* values are shown in the plots. PPV: positive predictive value; NPV: negative predictive value. **f**, **g** The ROC curves show the general performance of the DU_index in the NAcc (**f**) and the PFC (**g**).

Finally, we tried to find the general alterations of the methylation signature in both brain areas to distinguish illicit drug users from nonusers. We identified 35 cytosine sites (including 6 CpG sites) in the NAcc and 87 cytosine sites (including 15 CpG sites) in the PFC exhibiting differential methylation (*P* value < 0.05) between the control group and the drug use group using multivariable logistic regression (Fig. 4a–d). The details of those cytosine sites are listed in Additional file 2: Table S4 for the NAcc and Table S5 for the PFC. We constructed a drug-using index (DU_index) by summing the methylation levels of those cytosine sites using the logarithm of OR in each site as its weight. The DU_index score was significantly lower in the control group than in the drug use group in both the NAcc (p value = 4.70e−6, Fig. 4e) and the PFC (*P* value = 7.96e−8, Fig. 4e). Compared to the presence of illicit drug in blood tests, the DU_index score had a moderate (62% for the PFC) to low (29% for the NAcc) sensitivity but a perfect positive predictive value (100% for both the PFC and NAcc). The global performance of the DU_index score in distinguishing illicit drug users from nonusers was quite high, with an area under the ROC curves of 0.884 for the NAcc and 0.941 for the PFC (Fig. 4f, g).

### Methylation sites shared among indices

**Fig. 5:**
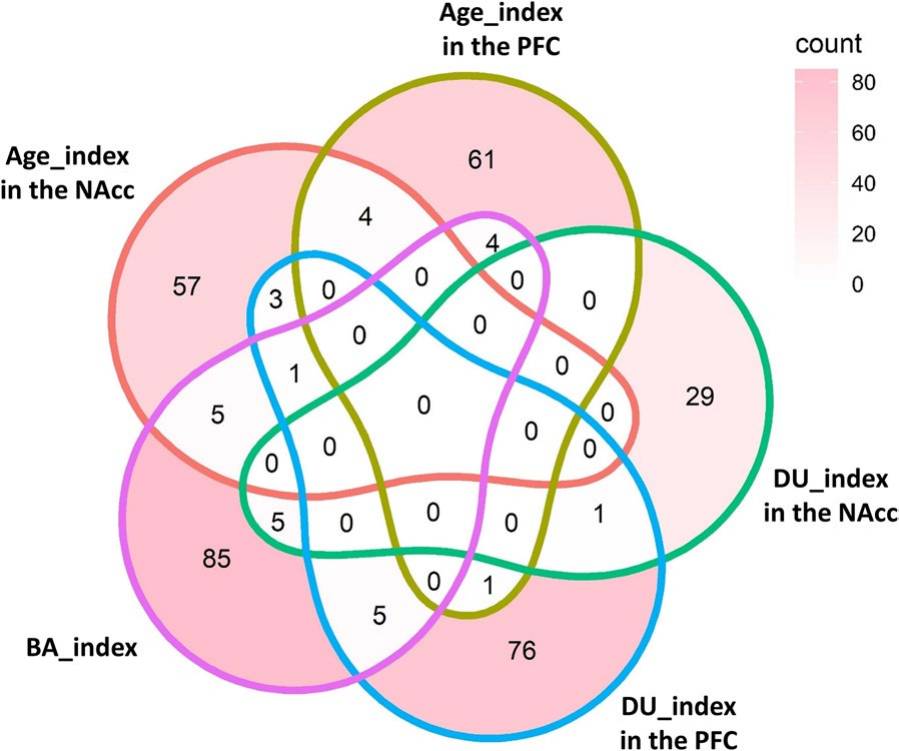
The selected cytosine sites in the BA_index, the Age_index, and the DU_index. The Venn diagram shows the selected cytosine sites in the different brain areas and the different indices under the *P* value threshold of 0.05.

For those cytosine sites selected in the three indices, the number of sites shared among different indices under the *P* value threshold of 0.05 is shown in Fig. 5. For example, there were 29 sites shared by any two indices, and 20 of them were components of the BA_index. Only a small number of cytosine sites, either in the Age_index (4 sites) or in the DU_index (1 site), were shared by the NAcc and the PFC.

### Sensitivity analysis

A series of sensitivity analyses was conducted over different stringencies of the *P* value threshold and the sequence contexts of cytosine. First, we changed the *P* value threshold from 0.05 to 0.005 for the BA_index score (Additional file 1: Figure S2) and the Age_index (Additional file 1: Figure S3) and from 0.05 to 0.02 for the DU_index (Additional file 1: Figure S4), and all the results remained similar. Second, we constructed individual index scores solely with either the CpG sites or the non-CpG sites for the BA_index, Age_index, and DU_index (Additional file 1: Figure S5). We found that both types of sequence context had similar performance except that the significance level of the index score consisting of non-CpG sites tended to be higher than the corresponding one of CpG sites, which was due to the higher proportion of non-CpG sites. Third, when the stringency of the *P* value threshold in constructing each index became more stringent (i.e., from 0.05 to 0.005 for BA_index and Age_index and to 002 for DU_index), no cytosine site was shared by any index (Additional file 1: Figure S6).

## Discussion

Mitochondrial DNA methylation has been the missing piece of the puzzle in epigenetics [6, 28, 29]. In this study, we first detected the methylation differences between the NAcc and the PFC and constructed a mitochondrial epigenetic clock using the association of aging with the methylation profile in the brain in the nonusers group and then found the effects of illicit drug use on the methylation levels in the lowering of the methylation levels in the NAcc but not in the PFC and age acceleration in the epigenetic clock, as compared to the nonusers group. Our findings suggest the importance of mitochondrial methylation in the human brain.

In our whole-genome bisulfite sequencing for mtDNA methylation, owing to the lower coverage on the L-strand than on the H-strand [21] and the stringent criterion of a coverage ≥ 30, the number of confirmed cytosine sites in the L-strand was reduced. Our findings of low methylation levels in the mitochondrial genome in the two brain areas (the NAcc and the PFC) were consistent with those of previous reports in different tissues [16, 17]. Unlike the predominance of CpG over non-CpG methylation in the nuclear genome [30], the average CpG and non-CpG methylation levels in the mitochondrial genome were almost equal. The utility of non-CpG methylation was further illustrated in the three indices constructed in this study with both non-CpG and CpG sites that had better distinction between brain areas, age groups, and the presence or absence of drug use than that consisting of the CpG sites only. Hence, the results of previous studies on the mitochondrial genome that were solely based on CpG sites [16, 17, 31, 32] should be interpreted cautiously.

Among the three indexes, the BA_index was constructed to reflect the specialized functions of the two brain areas in the control group by capturing the differential methylation signatures of the NAcc and the PFC. Intriguingly, the successful distinction by the BA_index is similar to that of a study using the differential methylation of the nuclear genome between the two brain areas [10], indicating that methylation in both mtDNA and nDNA is involved in the regulation of respective neuropsychiatric functions.

The Age_index, which was made up of the age-related cytosine sites in the mtDNA of the control group, exhibited positive correlations with chronological age, with an *R*^2^ ranging from 0.35 to 0.37. By comparing the prediction intervals between these brain areas, the epigenetic clock in the mitochondrial genome was consistently replicated in the two brain regions. One possibility for the correlation is due to the cycle theory that involves mitochondrial activity, mitochondrial DNA methylation, and alpha-ketoglutarate [33, 34]. As mitochondrial activity fades with aging, mitochondria gradually lose the ability to eliminate methylation on cytosines through alpha-ketoglutarate. Further investigation of the underlying mechanisms is warranted.

Using the Age_index to approximate the biological age, we further found that the presence of illicit drugs might accelerate the epigenetic age in the brain, particularly for ketamine. Ketamine is one of most commonly consumed novel psychoactive substances among the so-called club or party drugs [35], and its recreational use is common in Asia and has been associated with increased mortality, especially unnatural deaths [36]. Ketamine might change the epigenetic features of rat neurons through the effect of HDAC on BDNF [37] or the effect of NF-κB on COX-2 [38]. However, less is known about the influence of ketamine on the human mitochondrial genome. To our knowledge, this is the first report that ketamine might change the mitochondrial epigenetic clock in human brain tissues. In addition, the variation in age acceleration was wide in ATS users, resulting in the borderline significance of accelerated aging. Among the 9 ATS users, 6 used methamphetamine; therefore, the accelerated aging might be attributed to methamphetamine-induced senescence signs, e.g., greater loss of cortical gray matter than in nonusers [39].

Although the influence of illicit drugs could be detected through the use of the BA_index and the age index, the DU_index was constructed to summarize the general influence of illicit drugs on specific brain areas. Despite its low to moderate sensitivity, the DU_index had perfect specificity in both the NAcc and the PFC, resulting in an extremely high area under the curve (0.88 for the NAcc and 0.94 for the PFC). Although the sample size was small in this study, the almost perfect positive predictive value for the DU_index implies a potential application of the DU_index in detecting the use of ketamine or ATSs in forensic investigations.

Among the sites involved in different indices, we did find some sites shared between different indices but only when a less stringent P value threshold was adopted. Hence, it is plausible that the gene regulation via mitochondrial methylation to counteract the challenges from aging or illicit drugs in these two brain areas (the NAcc and the PFC) involves different cytosine sites.

## Limitations

This study has limitations. First, owing to the decomposition of postmortem brains from forensic cases, we analyzed bulk brain tissues instead of isolated neuronal cells and gained fewer reads from the NAcc than from the PFC. Second, some scheduled drugs were not analyzed in this study either because they were not included in our routine laboratory tests (such as marijuana) or because none of the specimens were positive for the drug (such as cocaine). Finally, despite the sample size of this study being larger than that of previous studies [23, 26], further validation from independent datasets of larger sample sizes is warranted to achieve the robustness in the prediction accuracy of the epigenetic clock in the mitochondrial genome like the epigenetic clock in the nuclear genome did [12, 13].

## Conclusion

In the human brain, we constructed three indices with the methylation levels of both the CpG and the non-CpG sites from the mitochondrial genome. Using the BA_index, reflecting the functional differences, we found that the BA_index scores were different between the NAcc and the PFC, and the diminished scores by illicit drugs in the NAcc implied the arousal of addiction behaviors. In addition, we examined the mitochondrial epigenetic clock through the Age_index and evaluated the age acceleration in deceased individuals with the presence of illicit drugs, especially ketamine. Finally, we summarized the general impact of illicit drugs on mitochondrial DNA methylation with the DU_index. Therefore, we showed the importance of mitochondrial epigenetics in the human brain, including the determination of specialized brain function, the estimation of biological age, and the identification of illicit drug users.

## Methods

### Sample collection

The study sample was selected from deceased individuals who were sent to the Institute of Forensic Medicine, Ministry of Justices in Taiwan for forensic investigation from August 21, 2018, to December 31, 2019, as depicted in Additional file 1: Figure S1. These forensic samples were available only by the employees of the institute for confidentiality concerns. Based on the information provided in the autopsy diary, the inclusion criteria for the study sample included male sex, being 18 years old or older, and no known major psychiatric disorder. We excluded those who died of drowning, burns, electrical shock, mechanical brain injuries, or heavy decomposition. For each included deceased individual, information about the cause of death and the results of toxicological analyses, including blood alcohol concentration, recent medical history reported by the prosecutor’s office, birth date, and postmortem interval (PMI), was also collected. One deceased individual without a toxicological report was assigned to the control group based on his medical history. Finally, we collected 105 brain tissues from 53 deceased individuals in this study. The sample size of the control group (*n* = 39 for each of the NAcc and the PFC) provided a statistical power of > 0.9 (alpha = 0.05) if the Pearson’s correlation r is over 0.5.

### Isolation of mitochondrion

After the left brain of each deceased individual was soaked in PBS to wash away blood, the meninges were stripped off, and the prefrontal cortex (PFC) and the nucleus accumbens (NAcc) (approximately 0.5 mg each) were further collected.

The protocol was slightly modified from that of Rueda C.B.[40]. Briefly, brain tissues (the PFC or the NAcc) were homogenized with a prechilled grinder. The homogenized tissues were treated with 5 mL mitochondrial isolation buffer and centrifuged at 700 rpm for 5 min at room temperature. The step was repeated once. Then, the supernatants were centrifuged at 7000 rpm for 5 min at room temperature, and the pellets were subjected to extraction for DNA using a PureLink Genomic DNA Mini Kit (Thermo Fisher, Waltham, MA, USA). The purified DNA was quantitated with Qubit4 (Thermo Fisher).

### Whole-mitochondrial-genome bisulfite sequencing

Approximately 100–300 ng of mitochondrial DNA was treated with Ovation Ultralow Methyl-Seq Library Systems (NuGEN, Redwood City, CA, USA) for the analysis of DNA methylation. Briefly, mitochondrial DNA was dissolved in nuclease-free water and fragmented to the appropriate length with Covaris E220 (Covaris, Woburn, MA, USA). The fragmented DNA was purified with Agencourt Ampure XP (Beckman Coulter, Brea, CA, USA) and dissolved in nuclease-free water. The purified fragmented DNA was subjected to end repair, ligation with a specific barcode, purification, and then final repair. The DNA was further denatured to expose the bases to bisulfite and then purified. The converted DNA was amplified with 15-cycle to 18-cycle PCR, purified again, and then served as a stock library. The library was quantitated with the KAPA Library Quantification Kit for Illumina® Platforms (KAPA Biosystems, Cape Town, South Africa). The sequencing platform was Illumina MiSeq FGx with MiSeq Reagent Kit v3 (Illumina, San Diego, CA, USA), which can provide reads with lengths up to 300 bps (single end).

An unmethylated amplicon serving as the negative control was subjected to the bisulfite sequencing in each batch. The bisulfite conversion rate of each batch was > 99%.

### Read quality check and alignment

The generated read 1 sequences were collected. The first 5 bases, the last 25 or 65 bases, and the low-quality bases (< Q30) were trimmed off (TrimGalore [41]) to retain the high-quality reads. The reads after quality check were aligned (Bismark [42]) using a high-sensitivity mode (-L 10 -D 20 -score_min l,0,-0.6) to the hg38 (NC_012920.1, containing NUMTs) with a modified mitochondrial genome, and then the mitochondrial genome was extracted with Samtools [43] (view -h ‘chrM’). The modified mitochondrial genome contained 4 extra Ns at each end to increase the alignment efficiency, and the reported cytosine sites were adjusted for the 4 Ns. The extracted bam files were analyzed with the MethylKit [44] package in R [45] for methylation level determination (percMethylation, filterByCoverage(lo.count = 30)), i.e., $\mathrm{methylation level}=100\times \left[{\mathrm{num}C}_{i}/\left({\mathrm{num}C}_{i}+{\mathrm{num}T}_{i}\right)\right]$, where num*C*_*i*_ = number of methylated *Cs* at the *i*th cytosine, num*T*_*i*_ = number of unmethylated *Cs* at the *i*th cytosine.

### Statistical analysis

The Chi-square test was used to analyze the distribution of alcohol intake, PMI, and cause of death between the control group and the drug use group. The Mann–Whitney U test was used to analyze the distribution of the age, the BA_index scores, the DU_index scores, and the age acceleration between different groups. The Wilcoxon signed rank test for matched samples was used to analyze the distribution of the methylation levels and the BA_index scores between the NAcc and the PFC of the same individual.

Cytosines with differential methylation between the NAcc and the PFC were used to compute a brain area index (BA_index) as follows: $\mathrm{BA}\_\mathrm{index}=\sum {\beta }_{i}\times {M}_{i}$, where ${\beta }_{i}$= log(fold change of NAcc over PFC at the ith cytosine), * M*_*i*_ = the methylation level (%) at the ith cytosine. Under this circumstance, a greater BA_index score means a higher methylation level.

For each brain area, the methylation levels in the control group and in the drug use group were compared using logistic regression in R, and the chronological age, experimental batches, doctors collecting tissues, alcohol intake, and PMI of each case were included as control variables (Additional file 2: Table S7). The PMI was dichotomized into ≤ 6 or > 6 h according to the practical acceptance of brain donation. Cytosines with differential methylation were used to compute a drug-using index (DU_index) as follows: $\mathrm{DU}\_\mathrm{index}=\sum {\beta }_{i}\times {M}_{i}$, where ${\beta }_{i}$= log(OR) at the ith cytosine from the logistic regression, and M_i_ = the methylation level (%) at the ith cytosine.

The threshold between controls and drug users was set as ${DA\_index}_{control}+1.96\times {SD}_{control}$, where DU_index_control_ is the averaged DU_index of controls and SD_control_ is the standard deviation of the DU_index of controls. The receiver operating characteristic curves were drawn with the “PRROC” package in R.

Pearson correlation was used to analyze the correlation between age and mitochondrial DNA methylation, and the experimental batches, doctors collecting tissues, alcohol intake, and PMI of each case were included as control variables. Cytosines with differential methylation were used to compute an Age_index as follows: $\mathrm{Age}\_\mathrm{index}=\sum {\beta }_{i}\times {M}_{i}$, where ${\beta }_{i}$= the slope at the ith cytosine from the Pearson correlation and M_i_ = the methylation level (%) at the ith cytosine. When each individual’s chronological age was regressed on the corresponding Age_index, the regression coefficient was used to calculate the predicted age. Age acceleration was defined as follows: age acceleration = predicted age − chronological age.

## Supplementary Information

**Additional file 1: Figure S1.** The collected samples of this study. **Figure S2**. The sensitivity analysis of BA_index. **Figure S3**. The sensitivity analysis of mitochondrial epigenetic clock. **Figure S4**. The sensitivity analysis of DU_index. **Figure S5**. The efficacy referred to the non-CpG sites and the CpG sites. **Figure S6**. The selected cytosine sites in the BA_index, the Age_index, and the DU_index.

**Additional file 2: Table S1.** Characteristics of the cases in the Drug use group by drug type and age. **Table S2**. The cytosine sites differentially methylated between the NAcc and the PFC. **Table S3**. The cytosine sites correlated with aging in the Nacc. **Table S4**. The cytosine sites correlated with aging in the PFC. **Table S5**. The cytosine sites differentially methylated between the control group and the drug use group in the Nacc. **Table S6**. The cytosine sites differentially methylated between the control group and the drug use group in the PFC. **Table S7**. The metadata of the deceased.

## Data Availability

The dataset supporting the conclusions of this article is available in the Sequence Read Archive (SRA) repository under BioProject number PRJNA763066. Data collection and analysis were not performed with blinding to the conditions of the experiments.

## Code availability

All codes for processing and analyzing the data presented in this work are available upon reasonable request.
