# Supplementary material for: Mitochondrial DNA methylation profiling of the human prefrontal cortex and nucleus accumbens: correlations with aging and drug use
Source: Clin Epigenetics. 2022 Jun 25;14:79. doi: 10.1186/s13148-022-01300-z (PMC9233363; doi:10.1186/s13148-022-01300-z)
Supplement: Supplementary file 1 — Additional file 1: Figure S1. The collected samples of this study. Figure S2. The sensitivity analysis of BA_index. Figure S3. The sensitivity analysis of mitochondrial epigenetic clock. Figure S4. The sensitivity analysis of DU_index. Figure S5. The efficacy referred to the non-CpG sites and the CpG sites. Figure S6. The selected cytosine sites in the BA_index, the Age_index, and the DU_index. [file 13148_2022_1300_MOESM1_ESM.docx]

**Additional file 1**

Figure S1. The collected samples of this study.

Figure S2. The sensitivity analysis of BA_index.

Figure S3. The sensitivity analysis of mitochondrial epigenetic clock.

Figure S4. The sensitivity analysis of DU_index.

Figure S5. The efficacy referred to the non-CpG sites and the CpG sites.

Figure S6. The selected cytosine sites in the BA_index, the Age_index, and the DU_index.


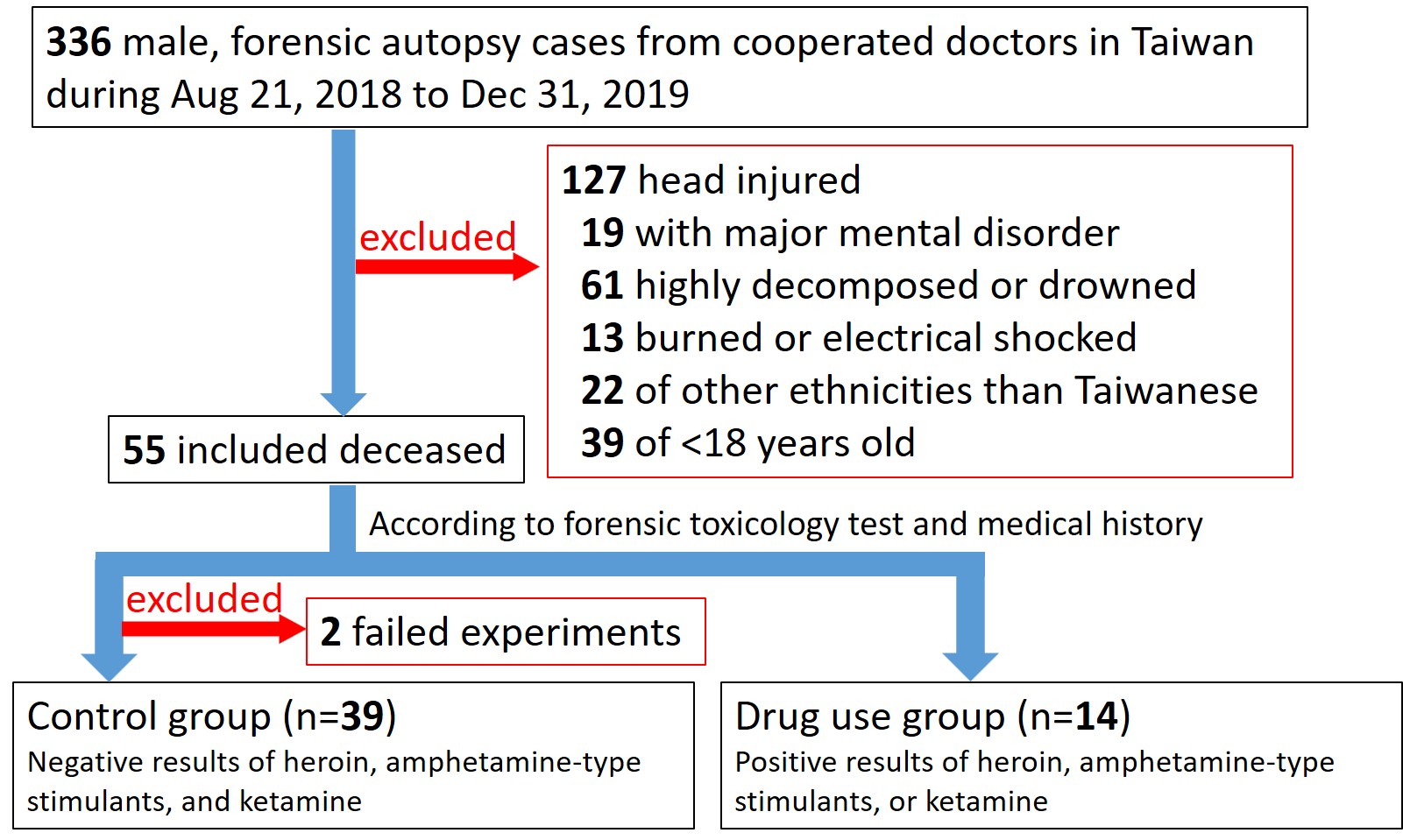


Figure S1. The collected samples of this study.

The cases were collected in Taiwan. The inclusion criteria were the male deceased individuals with age over 18 years old of cooperated doctors. The exclusion criteria were indicated in the plot. At last, we collected 39 deceased individuals for control group, and 14 deceased individuals for drug use group.


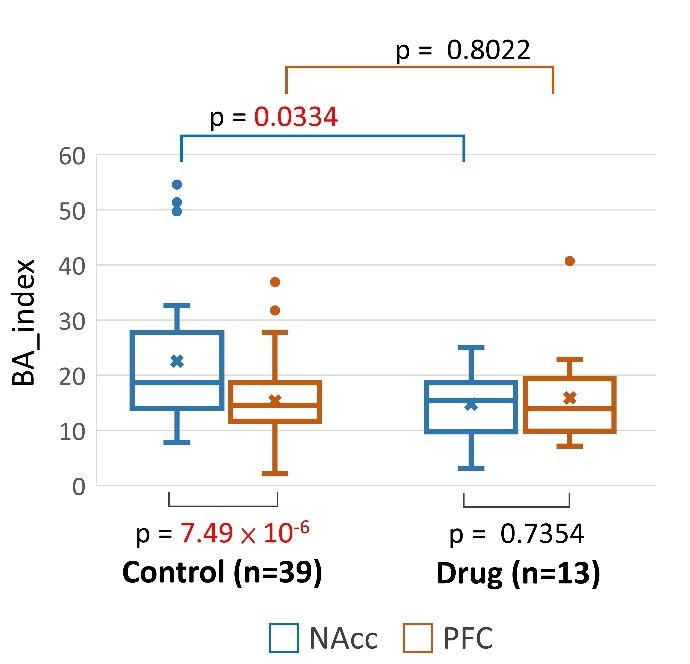


Figure S2. The sensitivity analysis of BA_index.

The box plot shows the distribution of BA_index with 12 cytosine sites (*P* < 0.005) in different brain areas and groups. The *P* values are shown in the plots.


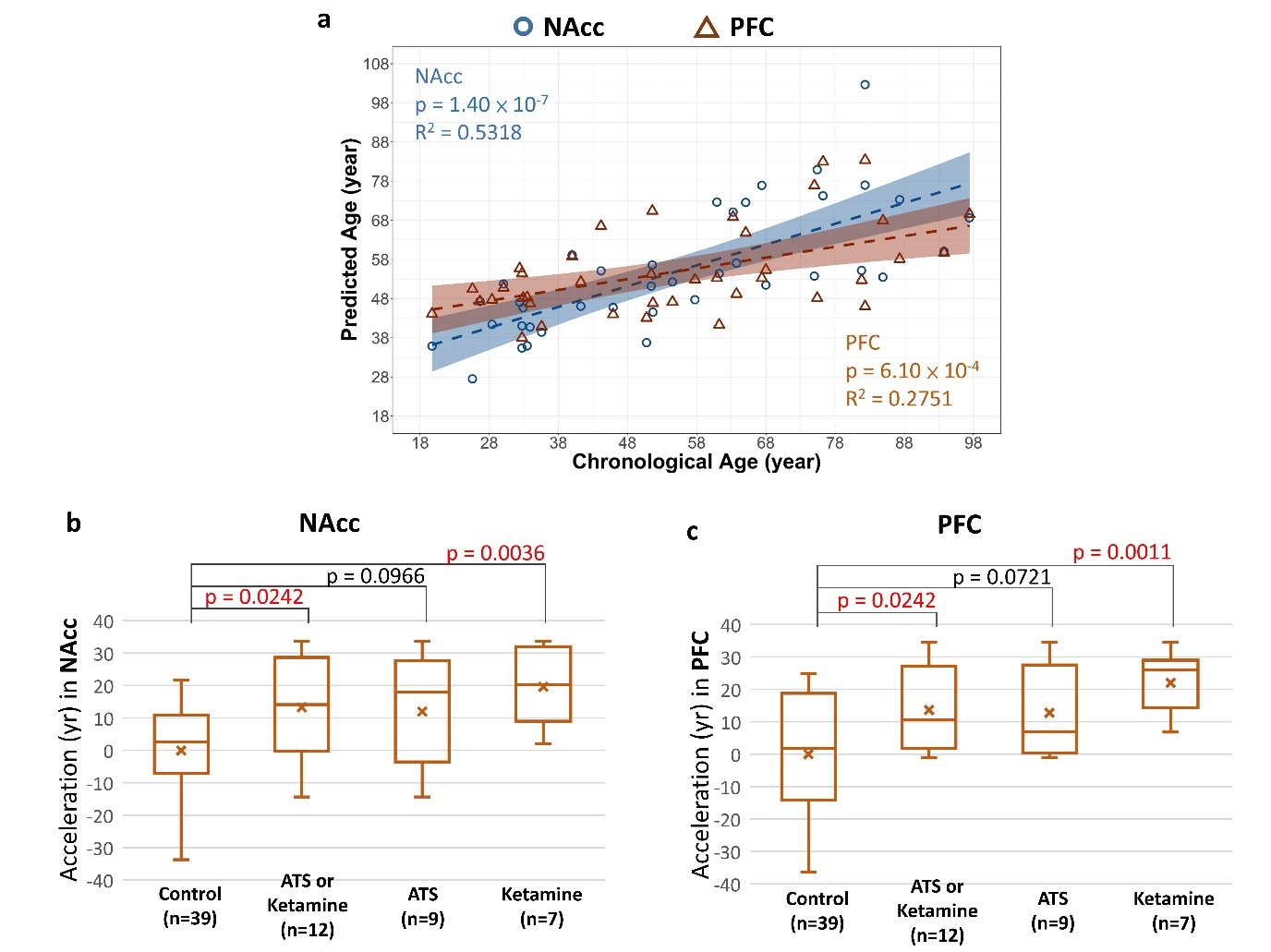


Figure S3. The sensitivity analysis of mitochondrial epigenetic clock.

For (**a**-**c**), under a stringent *P* value threshold of 0.005, the Age_index in the NAcc was composed of 6 cytosine sites and the Age_index in the PFC was composed of 2 cytosine sites. In **a**, the scatter plot shows the correlations between the chronological ages and the predicted ages from the NAcc (○) and the PFC (∆). The blue shade area (NAcc) and the brown shade area (PFC) represent the 95% confidence intervals for the predicted line in each region. The R^2^ and *P* value are indicated in the plot. In **b** and **c**, the box plots show the distribution of the accelerated ages in control group and different groups of illicit drugs in the NAcc and the PFC. The significance levels are shown in the plots.


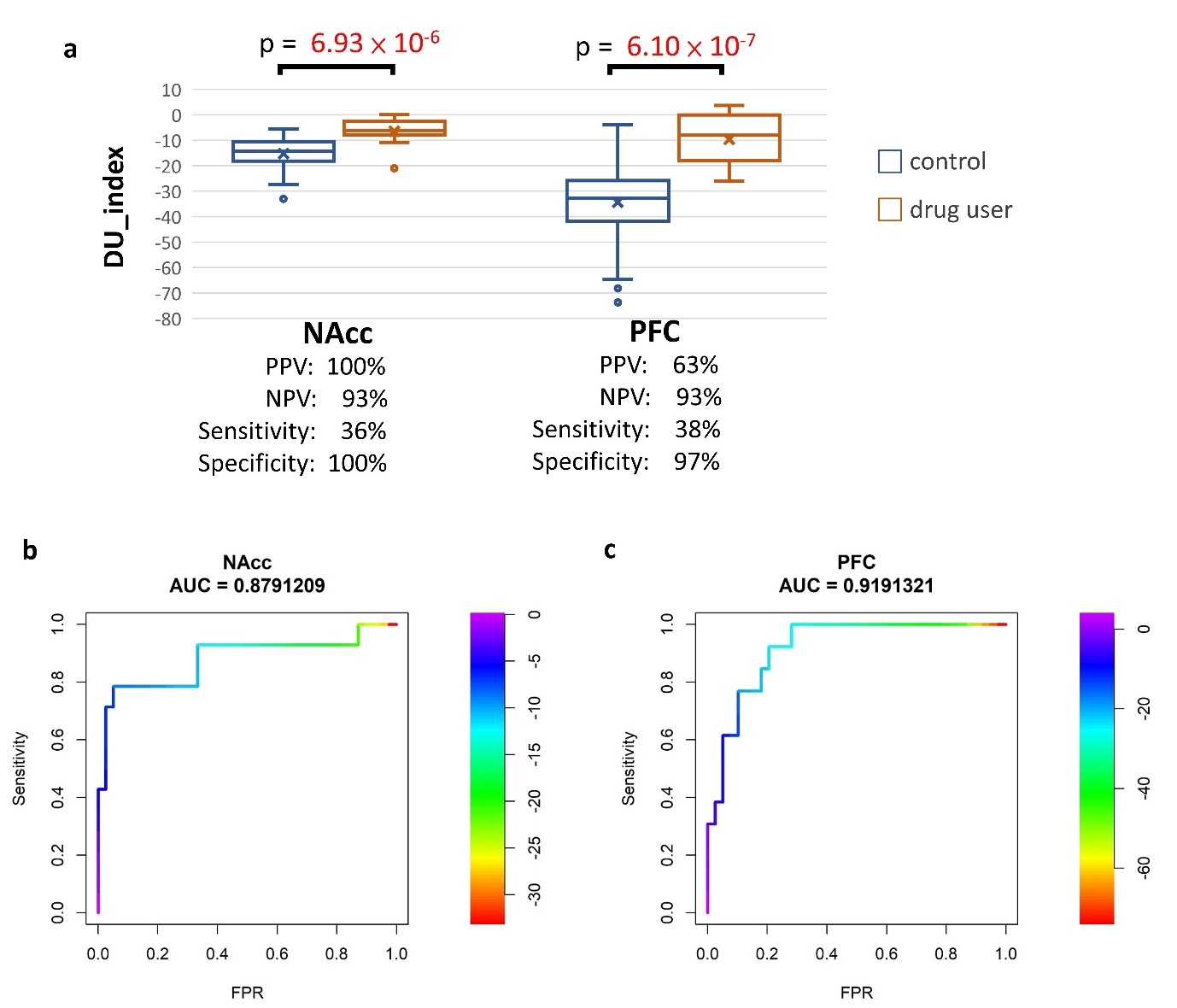


Figure S4. The sensitivity analysis of DU_index.

Under a stringent *P* value threshold of 0.02, the box plot (**a**) shows the distribution of the DU_index composed of 6 cytosine sites in the NAcc and the DU_index composed of 17 cytosine sites in the PFC. The *P* values are shown in the plots. PPV: positive predictive value; NPV: negative predictive value. The ROC curves show the general performance of the DU_index in the NAcc (**b**) and the PFC (**c**).


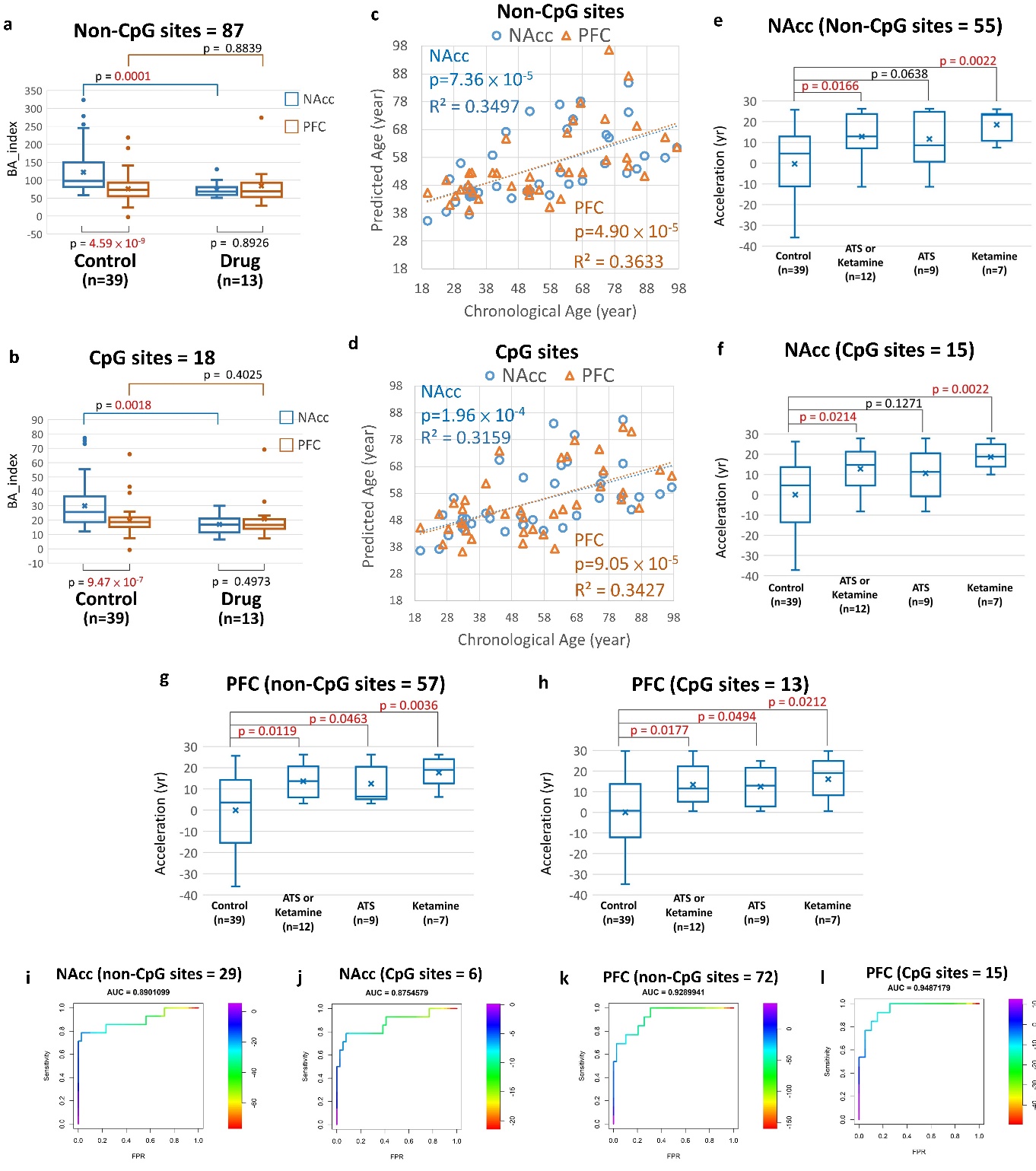


Figure S5. The efficacy referred to the non-CpG sites and the CpG sites.

We constructed the BA_index (**a**,**b**), Age_index (**c**-**h**), and the DU_index (**i**-**j**) with either non-CpG sites (**a**, **c**, **e**, **g**, **i**, and **k**) or the CpG sites (**b**, **d**, **f**, **h**, **j**, and **l**) with the P value threshold of 0.05. For **a** and **b**, the box plots show the distribution of BA_index in different brain areas and groups. For **c** and **d**, the scatter plots show the correlation between the chronological age and the predicted age. The brain areas, R^2^ and *P* value are indicated in the plot. For **e**-**h**, the box plots show the distribution of the accelerated ages in control group and different groups of illicit drugs in the NAcc and the PFC. The significance levels are shown in the plots. For **i**-**l**, the ROC curves show the general performance of the DU_index in the indicated brain areas. The quantities of the incorporated cytosines are indicated in the plots.

**
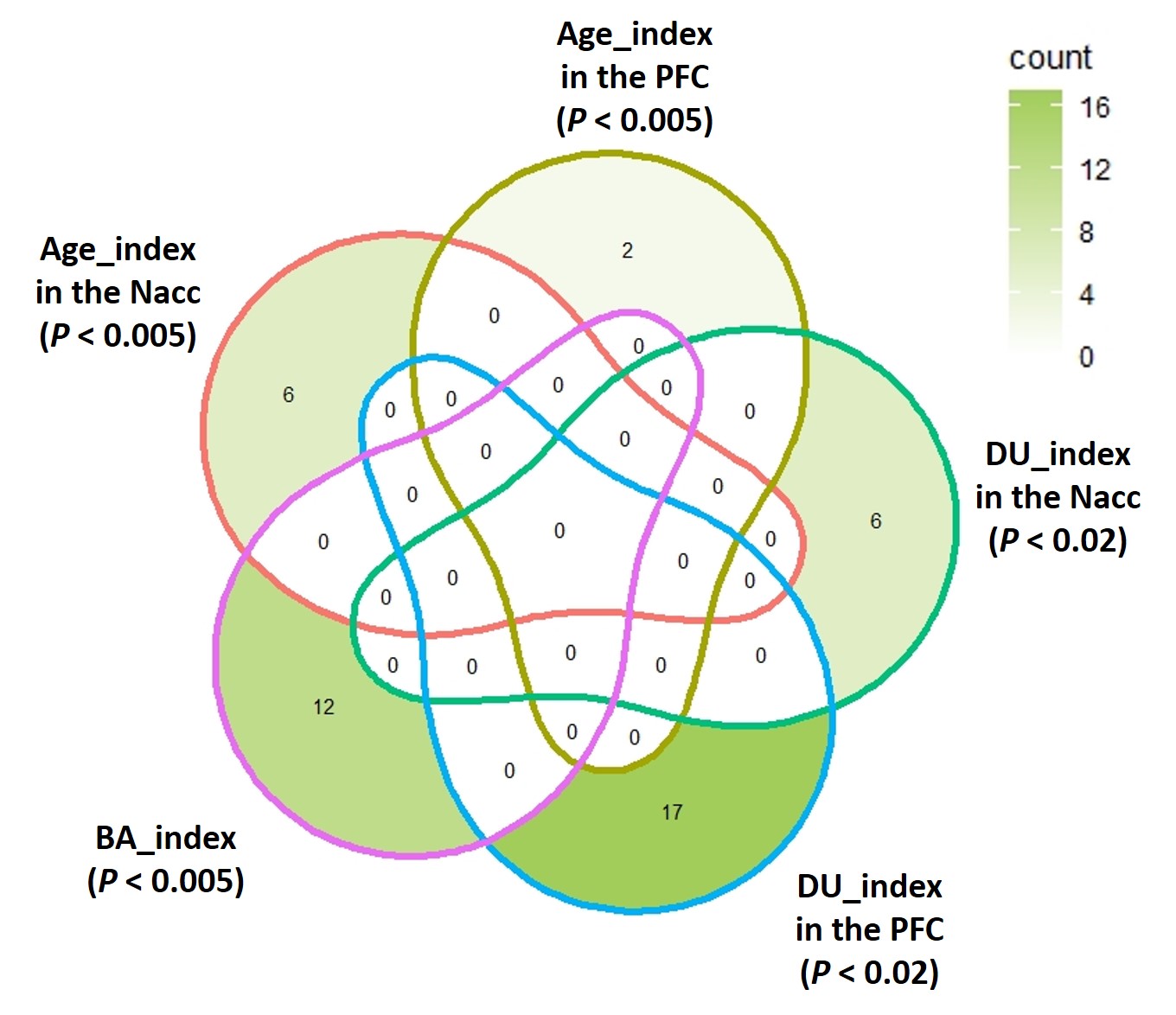
**

Figure S6. The selected cytosine sites in the BA_index, the Age_index, and the DU_index under the stringent *P* value threshold.

The Venn diagram shows the selected cytosine sites in the different brain areas and the different index systems under the stringent *P* value thresholds indicated in the plot.
