# Supplementary material for: Mitochondrial DNA methylation profiling of the human prefrontal cortex and nucleus accumbens: correlations with aging and drug use
Source: Clin Epigenetics. 2022 Jun 25;14:79. doi: 10.1186/s13148-022-01300-z (PMC9233363; doi:10.1186/s13148-022-01300-z)
Supplement: Supplementary file 2 — Additional file 2: Table S1. Characteristics of the cases in the Drug use group by drug type and age. Table S2. The cytosine sites differentially methylated between the NAcc and the PFC. Table S3. The cytosine sites correlated with aging in the Nacc. Table S4. The cytosine sites correlated with aging in the PFC. Table S5. The cytosine sites differentially methylated between the control group and the drug use group in the Nacc. Table S6. The cytosine sites differentially methylated between the control group and the drug use group in the PFC. Table S7. The metadata of the deceased. [file 13148_2022_1300_MOESM2_ESM.pdf]

Table S1. Characteristics of the cases in the Drug use group by drug type and age.

Case no.	Drug type	Age (yr)	Cause of death
8[1]	Heroin	39.8	Accident
16	Heroin	51.3	Accident
47	ATS[2](Methamphetamine)	37.8	Undetermined
28	ATS (Methamphetamine)	38.2	Accident
18	ATS (Methamphetamine)	41.1	Suicide
15	ATS (Methamphetamine)	54.9	Homicide
5	ATS (Methamphetamine)	59.6	Accident
24	ATS (Methamphetamine), Ketamine	21.1	Suicide
17	ATS (MDMA), Ketamine	24.2	Suicide
45	ATS (Mephedrone), Ketamine	29.6	Homicide
57	ATS (Mephedrone), Ketamine	40.8	Homicide
44	Ketamine	20.3	Homicide
58	Ketamine	30.4	Natural death
20	Ketamine	35.3	Homicide

[1] No adequate methylation data of PFC.

[2] Amphetamine-type stimulants.

Table S2. The cytosine sites differentially methylated between the NAcc and the PFC.

Position	Context	Gene	mean FoldChange log2(NAcc/PFC)	P value
14960	CHH	MT-CYB	2.534675	0.000105
13063	CHG	MT-ND5	1.248118	0.000302
15240	CHG	MT-CYB	2.422021	0.000559
9982	CHH	MT-CO3	-0.754208	0.001733
2943	CpG	MT-RNR2	0.576303	0.002894
12533	CHG	MT-ND5	2.425097	0.003092
13417	CHH	MT-ND5	2.298797	0.003894
3238	CHH	MT-TL1	-0.921570	0.003902
13048	CHH	MT-ND5	0.942288	0.004289
3230	CHH	MT-TL1	-0.153924	0.004685
13019	CHH	MT-ND5	0.485501	0.004719
11887	CHG	MT-ND4	1.694087	0.004949
10680	CHH	MT-ND4L	-2.446901	0.006540
15221	CHG	MT-CO2	1.765965	0.007492
7697	CHH	MT-CYB	2.441824	0.007492
15798	CHH	MT-CYB	2.237583	0.007956
13042	CHG	MT-ND5	0.432649	0.008191
12613	CHH	MT-ND5	0.465689	0.008667
2847	CHG	MT-RNR2	0.553675	0.009056
14655	CHG	MT-CO3	2.400005	0.009434
9932	CHH	MT-ND6	2.496524	0.009434
2478	CHG	MT-RNR2	1.846079	0.009763
3255	CpG	MT-TL1	-0.224689	0.009763
13031	CHG	MT-TM	0.386979	0.010643
4407	CHH	MT-ND5	0.352742	0.010643
13361	CHH	MT-ND5	0.958115	0.011591
9952	CHH	MT-CO3	1.790314	0.011623
4966	CHG	MT-ND2	-4.271878	0.012611
2477	CpG	MT-RNR2	-0.248796	0.012617
2889	CHH	MT-RNR2	1.155983	0.012617
12610	CHG	MT-ND5	2.887912	0.013149
10971	CHG	MT-ND1	1.653601	0.013707
3437	CHG	MT-ND4	-1.003146	0.013707
12763	CpG	MT-ND5	1.067278	0.014285
15215	CHH	MT-CYB	0.353558	0.014285
10360	CHH	MT-CO1	-1.516135	0.014884
6517	CHH	MT-CO2	1.821840	0.014884
7793	CpG	MT-ND3	1.125814	0.014884
15959	CHG	MT-RNR2	-1.598373	0.016453
2608	CHH	MT-TP	1.755912	0.016453
1302	CpG	MT-RNR1	3.002008	0.016812
3664	CHG	MT-ND1	-0.777330	0.016812
9986	CHH	MT-CO3	1.238423	0.018213
5293	CHH	MT-ND2	0.343021	0.018805
13018	CHH	MT-ND5	2.443149	0.018950
11832	CHH	MT-ND4	-0.297421	0.019550
12406	CpG	MT-ND5	1.644258	0.019713
687	CHH	MT-RNR1	0.329352	0.020501

Table S2. The cytosine sites differentially methylated between the NAcc and the PFC.

Position	Context	Gene	mean FoldChange log2(NAcc/PFC)	P value
9253	CHH	MT-CO3	1.025250	0.020501
12508	CHH	MT-ND5	1.054785	0.021841
2849	CHG	MT-RNR2	0.502952	0.021943
13399	CHH	MT-ND5	2.937047	0.022158
1018	CHH	MT-RNR1	2.885681	0.022725
12772	CHH	MT-ND5	0.955377	0.023028
13368	CHH	MT-ND5	3.014761	0.023028
14438	CHG	MT-ND6	0.430429	0.023028
12147	CHH	MT-CO2	0.409010	0.023927
15059	CpG	MT-TH	1.678780	0.023927
15217	CHH	MT-CYB	1.068807	0.023927
7637	CHH	MT-CYB	0.979105	0.023927
15995	CHH	MT-TP	0.351518	0.024586
13415	CHH	MT-ND5	2.222481	0.025814
7236	CpG	MT-CO1	0.933904	0.026497
10731	CHH	MT-TL1	-0.376625	0.026804
3277	CHG	MT-ND1	-0.893526	0.026804
3591	CHG	MT-CO3	0.277994	0.026804
9985	CHH	MT-ND4L	-0.164031	0.026804
9394	CpG	MT-CO3	-0.193193	0.027500
7640	CHH	MT-CO2	0.946887	0.027825
2116	CHH	MT-RNR2	-4.278887	0.028879
9247	CHG	MT-CO3	2.294096	0.028879
12814	CpG	MT-ATP6	0.915894	0.029967
9182	CHH	MT-ND5	1.065786	0.029967
9379	CHH	MT-CO3	-0.254242	0.030708
12517	CHH	MT-ND5	-1.049504	0.031846
4491	CpG	MT-ND2	1.742823	0.032173
3688	CpG	MT-ND1	1.608491	0.033436
2100	CHH	MT-RNR2	1.044189	0.033481
15894	CHH	MT-TT	0.992856	0.034232
15777	CHH	MT-CYB	0.987967	0.035481
2877	CpG	MT-RNR2	1.710550	0.035930
3226	CHH	MT-RNR2	2.365748	0.035962
13069	CHG	MT-ND1	3.114718	0.037235
3697	CpG	MT-ND5	1.079706	0.037235
8153	CHG	MT-CO2	-3.637100	0.037351
10472	CHH	MT-ND4L	2.283901	0.038096
15797	CHH	MT-CYB	1.012841	0.038578
12871	CpG	MT-ND5	-1.049435	0.039961
769	CHG	MT-RNR1	1.626240	0.040342
2844	CpG	MT-RNR2	2.271105	0.041384
8187	CHH	MT-CO2	1.030219	0.041899
11414	CHH	MT-ND4	1.562436	0.042850
11518	CHG	MT-ND4	-0.466364	0.042850
12226	CHG	MT-TS2	1.615493	0.042850
658	CHH	MT-RNR1	1.078407	0.043819
10662	CHH	MT-CO2	-2.959321	0.044358

Table S2. The cytosine sites differentially methylated between the NAcc and the PFC.

Position	Context	Gene	mean FoldChange log2(NAcc/PFC)	P value
8219	CHH	MT-ND4L	0.451397	0.044358
1007	CHH	MT-RNR1	2.879383	0.044809
6537	CHG	MT-CO1	0.385805	0.045359
10677	CpG	MT-ND4L	-2.286275	0.045909
13085	CHH	MT-ND5	1.679298	0.045909
11346	CHH	MT-ND4	0.998266	0.047505
15246	CHH	MT-CYB	0.367104	0.047505
12372	CHG	MT-ND5	-1.002590	0.048120
13477	CHH	MT-ND5	0.301089	0.049146

Table S3. The cytosine sites correlated with aging in the Nacc.

Position	Context	Gene	slope	P value
2655	CHG	MT-RNR2	191.712305	0.002612
5353	CpG	MT-ND2	103.413259	0.002976
15043	CHH	MT-CYB	188.317080	0.003089
877	CHH	MT-RNR1	145.381575	0.003264
11226	CHH	MT-ND4	100.337045	0.003986
3538	CHH	MT-ND1	101.326788	0.004539
2435	CHG	MT-RNR2	106.579818	0.005849
878	CHH	MT-RNR1	118.175208	0.005854
11196	CHH	MT-ND4	139.599503	0.006337
7757	CpG	MT-CO2	138.547547	0.006949
9142	CHG	MT-ATP6	112.202771	0.007836
3745	CHH	MT-ND1	130.164296	0.007844
11150	CHH	MT-ND4	125.004041	0.008537
15217	CHH	MT-CYB	108.959260	0.010472
9253	CHH	MT-CO3	99.988934	0.010650
8152	CpG	MT-CO2	125.516940	0.012441
3550	CpG	MT-ND1	98.614397	0.012788
2174	CHH	MT-RNR2	145.148244	0.012953
12192	CpG	MT-TH	98.037953	0.014305
14869	CHG	MT-CYB	73.084565	0.015466
3850	CHH	MT-ND1	116.657201	0.016685
3315	CHH	MT-ND1	99.357768	0.017163
15216	CHH	MT-CYB	96.759935	0.017337
15221	CHG	MT-CYB	92.253281	0.017505
11963	CHH	MT-ND4	78.504867	0.017931
3882	CHH	MT-ND1	88.900004	0.018733
2068	CHH	MT-RNR2	150.907633	0.019303
9025	CHG	MT-ATP6	118.867978	0.019980
9026	CHH	MT-ATP6	113.937700	0.020006
3698	CHG	MT-ND1	92.736418	0.021131
3424	CHH	MT-ND1	87.704629	0.021517
13590	CHG	MT-ND5	108.205599	0.022775
11225	CHH	MT-ND4	107.793088	0.022835
6541	CpG	MT-CO1	75.658440	0.023026
11184	CpG	MT-ND4	122.392286	0.024573
15005	CpG	MT-CYB	133.032867	0.025646
11176	CHG	MT-ND4	150.028472	0.026661
3922	CpG	MT-ND1	94.877244	0.027390
15240	CHG	MT-CYB	86.319916	0.027439
5460	CpG	MT-ND2	103.176472	0.027759
7779	CHG	MT-CO2	92.219915	0.028522
3849	CHH	MT-ND1	97.005552	0.028678
2070	CHH	MT-RNR2	143.315456	0.029953
7789	CHG	MT-CO2	91.149968	0.031334
8517	CHG	MT-ATP8	105.395742	0.031434

Table S3. The cytosine sites correlated with aging in the Nacc.

Position	Context	Gene	slope	P value
3877	CHH	MT-ND1	106.221977	0.031977
9266	CHH	MT-CO3	101.779514	0.033360
3412	CHH	MT-ND1	72.942641	0.033680
664	CHH	MT-RNR1	92.188088	0.033951
658	CHH	MT-RNR1	76.978958	0.035844
7664	CpG	MT-CO2	79.849276	0.036244
12185	CHH	MT-TH	69.541794	0.037578
3776	CHH	MT-ND1	78.227952	0.037974
3531	CpG	MT-ND1	47.971175	0.039354
3693	CHG	MT-ND1	74.937292	0.039386
6967	CHH	MT-CO1	112.806823	0.039826
12183	CHG	MT-TH	71.734967	0.040789
13288	CpG	MT-ND5	102.241298	0.041405
5828	CHH	MT-TY	99.960671	0.043993
872	CHG	MT-RNR1	105.756736	0.044296
4376	CpG	MT-TQ	71.887274	0.045020
14861	CpG	MT-CYB	60.320604	0.045099
13268	CHH	MT-ND5	104.021363	0.046530
3665	CHH	MT-ND1	65.502290	0.046849
9182	CHH	MT-ATP6	72.534625	0.046967
2976	CHH	MT-RNR2	94.764700	0.047128
2421	CHG	MT-RNR2	58.308128	0.047551
10704	CHH	MT-ND4L	95.331087	0.048258
3842	CHH	MT-ND1	74.633382	0.048388
11222	CHH	MT-ND4	106.609833	0.048934

Table S4. The cytosine sites correlated with aging in the PFC.

Position	Context	Gene	slope	P value
2684	CHG	MT-RNR2	168.765377	0.000526
8921	CHH	MT-ATP6	116.713437	0.001168
6363	CHG	MT-CO1	215.377896	0.005551
10266	CHH	MT-ND3	139.992093	0.006121
3357	CHH	MT-ND1	89.235406	0.007237
15356	CHG	MT-CYB	168.621275	0.008555
3877	CHH	MT-ND1	114.234812	0.012269
4812	CHH	MT-ND2	111.993646	0.012673
3913	CHH	MT-ND1	111.193047	0.013427
8873	CHH	MT-ATP6	110.971437	0.013839
11559	CHH	MT-ND4	101.027136	0.014082
3358	CHH	MT-ND1	87.911714	0.014475
3235	CHH	MT-TL1	108.331948	0.014598
8920	CHH	MT-ATP6	92.682785	0.015162
3427	CHH	MT-ND1	81.602840	0.015827
2170	CHH	MT-RNR2	101.596529	0.017635
2788	CHH	MT-RNR2	-139.203711	0.018447
1361	CHH	MT-RNR1	84.508048	0.018501
3424	CHH	MT-ND1	75.155997	0.018705
13001	CHH	MT-ND5	108.595805	0.020331
3352	CpG	MT-ND1	84.051114	0.020339
2996	CHH	MT-RNR2	111.944900	0.021946
12821	CHG	MT-ND5	-145.941396	0.023751
6383	CHH	MT-CO1	115.130011	0.024157
10522	CHH	MT-ND4L	101.246930	0.025497
8581	CpG	MT-ATP6	123.031358	0.026997
3882	CHH	MT-ND1	82.263995	0.027267
11408	CHH	MT-ND4	72.739092	0.027676
3337	CHH	MT-ND1	80.419282	0.028649
10662	CHH	MT-ND4L	92.941071	0.028730
3014	CHH	MT-RNR2	104.038152	0.029684
11407	CHH	MT-ND4	92.976559	0.029696
5224	CHH	MT-ND2	73.128289	0.029998
11396	CHH	MT-ND4	94.540790	0.030051
8856	CpG	MT-ATP6	92.209491	0.030121
4794	CHH	MT-ND2	76.735203	0.030344
2836	CHH	MT-RNR2	-104.164426	0.032823
10143	CpG	MT-ND3	93.178658	0.033263
2688	CHG	MT-RNR2	114.646256	0.033473
10023	CHH	MT-TG	54.435245	0.033528
10677	CpG	MT-ND4L	78.193638	0.034998
3659	CHG	MT-ND1	113.975539	0.037969
11546	CHH	MT-ND4	109.581996	0.038319
3316	CHH	MT-ND1	75.058304	0.038734
1336	CHH	MT-RNR1	72.325673	0.040016

Table S4. The cytosine sites correlated with aging in the PFC.

Position	Context	Gene	slope	P value
6166	CHG	MT-CO1	58.382521	0.040747
10671	CpG	MT-ND4L	98.905268	0.040918
4563	CHH	MT-ND2	-120.235027	0.041029
3907	CHH	MT-ND1	104.545487	0.041649
2915	CpG	MT-RNR2	91.645428	0.041690
7197	CpG	MT-CO1	130.567497	0.042132
9445	CpG	MT-CO3	117.530616	0.042570
8392	CHH	MT-ATP8	100.944330	0.043734
11414	CHH	MT-ND4	88.202210	0.043980
8969	CHG	MT-ATP6	82.835485	0.044266
8989	CHH	MT-ATP6	76.985015	0.044386
10683	CHG	MT-ND4L	98.525302	0.045494
4378	CHH	MT-TQ	57.764292	0.045928
11657	CHG	MT-ND4	130.286374	0.046609
12876	CpG	MT-ND5	46.131835	0.047071
3526	CpG	MT-ND1	57.772156	0.047457
9002	CpG	MT-ATP6	115.142650	0.047567
12997	CHG	MT-ND5	104.305140	0.047968
10230	CHH	MT-ND3	82.674709	0.048224
1345	CHH	MT-RNR1	84.999680	0.048274
12891	CHH	MT-ND5	49.434654	0.048684
2847	CHG	MT-RNR2	-149.887075	0.048839
3849	CHH	MT-ND1	81.780477	0.049006
3901	CpG	MT-ND1	87.822231	0.049501
3001	CHG	MT-RNR2	77.740940	0.049552

Table S5. The cytosine sites differentially methylated between the control group and the drug use group in the Nacc.

Position	Context	Gene	log(OR)	P value
15045	CpG	MT-CYB	-1.902307	0.009206
9329	CpG	MT-CO3	-1.424542	0.010090
10998	CHH	MT-ND4	-0.977218	0.011662
2735	CHH	MT-RNR2	-1.646361	0.012577
12186	CHH	MT-TH	-1.840291	0.016137
15731	CHH	MT-CYB	-1.631249	0.019758
10646	CHH	MT-ND4L	1.035990	0.022119
6909	CHG	MT-CO1	-1.256600	0.022824
15500	CpG	MT-CYB	-1.137258	0.022918
1247	CHH	MT-RNR1	-1.372070	0.025172
9715	CHH	MT-CO3	-1.240558	0.025886
6513	CHG	MT-CO1	-1.018119	0.026844
15317	CHG	MT-CYB	-0.986101	0.031009
7198	CHG	MT-CO1	-1.197759	0.033005
9663	CHH	MT-CO3	-0.557318	0.033772
13368	CHH	MT-ND5	-1.328611	0.034311
9382	CpG	MT-CO3	-1.141040	0.034527
5821	CpG	MT-TC	-1.433892	0.034541
2849	CHG	MT-RNR2	-0.887681	0.034617
11891	CHH	MT-ND4	0.916201	0.034695
10994	CHH	MT-ND4	-0.702439	0.035854
6931	CHH	MT-CO1	-1.054660	0.036189
2067	CHH	MT-RNR2	0.766073	0.036225
3666	CHH	MT-ND1	-1.029127	0.036861
2877	CpG	MT-RNR2	-1.259468	0.039158
8187	CHH	MT-CO2	-1.414488	0.039413
6504	CHG	MT-CO1	-0.999714	0.040080
6516	CHG	MT-CO1	-0.769162	0.040285
9690	CHH	MT-CO3	-0.980203	0.041057
1278	CHH	MT-RNR1	0.705902	0.041058
10695	CHH	MT-ND4L	1.049654	0.041369
15215	CHH	MT-CYB	-1.103971	0.041400
10993	CHH	MT-ND4	-0.584246	0.042977
13730	CHH	MT-ND5	-1.194738	0.046980
11016	CHG	MT-ND4	-0.556497	0.048686

Table S6. The cytosine sites differentially methylated between the control group and the drug use group in the PFC.

Position	Context	Gene	log(OR)	P value
15926	CpG	MT-TT	-2.782649	0.006109
1474	CpG	MT-RNR1	-2.775811	0.007182
785	CpG	MT-RNR1	3.790019	0.007961
7928	CpG	MT-CO2	-2.709109	0.008239
1350	CHH	MT-RNR1	-1.769737	0.009507
9715	CHH	MT-CO3	-2.238095	0.009633
8998	CpG	MT-ATP6	-2.335727	0.010046
10375	CHH	MT-ND3	1.984035	0.011339
2236	CHH	MT-RNR2	-2.179149	0.015354
9628	CHH	MT-CO3	-1.741051	0.015721
1910	CHH	MT-RNR2	2.211924	0.015949
3712	CHG	MT-ND1	-2.766741	0.017162
13204	CHG	MT-ND5	-1.964700	0.018025
3437	CHG	MT-ND1	-1.653585	0.018535
9295	CHG	MT-CO3	-1.472496	0.018958
6182	CpG	MT-CO1	-1.223466	0.019047
7467	CHH	MT-TS1	1.630909	0.019069
12294	CHH	MT-TL2	-2.423222	0.020387
12763	CpG	MT-ND5	2.518802	0.020833
1030	CHH	MT-RNR1	1.103853	0.021820
12962	CHH	MT-ND5	-1.714890	0.022096
2546	CHG	MT-RNR2	-2.557151	0.022417
1290	CHH	MT-RNR1	-2.927014	0.022666
2695	CHH	MT-RNR2	-2.127394	0.022730
10726	CHH	MT-ND4L	-1.379262	0.022955
3698	CHG	MT-ND1	-2.211753	0.023124
3718	CHH	MT-ND1	1.130750	0.023691
15216	CHH	MT-CYB	-2.143758	0.024078
1923	CHH	MT-RNR2	1.669285	0.024229
15240	CHG	MT-CYB	-1.520491	0.025287
9621	CpG	MT-CO3	-1.315895	0.025663
2700	CpG	MT-RNR2	-2.318352	0.026316
2867	CHH	MT-RNR2	-1.771011	0.027430
15439	CHH	MT-CYB	1.575756	0.027515
12946	CHH	MT-ND5	-1.765767	0.027875
9276	CHG	MT-CO3	-1.938608	0.028114
1930	CHG	MT-RNR2	1.993321	0.028402
10360	CHH	MT-ND3	1.627151	0.028574
15945	CHH	MT-TT	1.637331	0.028617
15346	CHH	MT-CYB	-2.078918	0.029006
3607	CHH	MT-ND1	-1.731034	0.030671
14439	CHH	MT-ND6	1.061583	0.030851
1748	CHH	MT-RNR2	-1.980682	0.030954
11274	CHH	MT-ND4	-1.482117	0.030976

Table S6. The cytosine sites differentially methylated between the control group and the drug use group in the PFC.

Position	Context	Gene	log(OR)	P value
12929	CHH	MT-ND5	-1.397422	0.031116
13031	CHG	MT-ND5	0.957055	0.031151
15356	CHG	MT-CYB	-2.389609	0.031289
9962	CHG	MT-CO3	1.484173	0.031787
904	CHH	MT-RNR1	-1.885682	0.031933
10407	CHH	MT-TR	1.371768	0.032865
11478	CHG	MT-ND4	-1.853610	0.033116
12533	CHG	MT-ND5	1.600071	0.033466
2400	CHH	MT-RNR2	0.744453	0.033843
2771	CHH	MT-RNR2	-1.602775	0.035000
3915	CHH	MT-ND1	-2.008851	0.035387
3438	CHH	MT-ND1	-1.273326	0.035880
1179	CHG	MT-RNR1	-1.276065	0.035956
12816	CHG	MT-ND5	-1.793651	0.036355
2019	CHH	MT-RNR2	-1.577382	0.037247
10144	CHG	MT-ND3	-1.507424	0.038172
4333	CHH	MT-TQ	1.569753	0.038285
2397	CHH	MT-RNR2	0.795473	0.038835
2698	CHH	MT-RNR2	-1.711323	0.039067
15173	CHH	MT-CYB	-1.314334	0.039294
15152	CHH	MT-CYB	-1.248503	0.039918
1833	CHH	MT-RNR2	1.733785	0.040710
7287	CHH	MT-CO1	1.059915	0.041100
14226	CpG	MT-ND6	1.374925	0.041218
15452	CHH	MT-CYB	0.952848	0.041437
6180	CHH	MT-CO1	-1.121037	0.041988
626	CHG	MT-TF	-1.251900	0.042640
1476	CpG	MT-RNR1	-1.985890	0.042726
3918	CHH	MT-ND1	-1.688908	0.042838
11561	CHH	MT-ND4	-1.171793	0.043389
7984	CHG	MT-CO2	1.107595	0.043450
6618	CpG	MT-CO1	-1.256818	0.043589
7164	CpG	MT-CO1	0.953957	0.043699
8126	CHH	MT-CO2	-1.175517	0.043785
3436	CpG	MT-ND1	-1.051309	0.045583
15153	CHH	MT-CYB	-1.433824	0.045699
7327	CHH	MT-CO1	1.124382	0.047046
5140	CHG	MT-ND2	-1.177871	0.047645
3693	CHG	MT-ND1	-1.238827	0.047708
12756	CHG	MT-ND5	2.946916	0.047816
5226	CHH	MT-ND2	0.595517	0.048049
627	CHH	MT-TF	-1.577229	0.048194
7341	CpG	MT-CO1	1.478544	0.048568

Table S7. The metadata of the deceased.

Case no.	drug use group	Batch	Doctor collecting tissue	Alcohol intake	Postmortem interval >6 hours
2	No	A	B	Yes	No
3	No	A	B	No	No
4	No	A	B	Yes	Yes
5	Yes	A	A	No	No
6	No	A	A	No	Yes
7	No	A	A	No	No
8	Yes	A	B	Yes	No
9	No	A	B	Yes	No
10	No	A	A	No	No
11	No	A	A	No	No
12	No	A	B	No	No
13	No	A	B	No	No
14	No	A	B	No	No
15	Yes	A	A	No	No
16	Yes	B	B	No	Yes
17	Yes	B	A	No	Yes
18	Yes	B	A	No	No
19	No	B	A	No	No
20	Yes	B	A	No	Yes
21	No	B	A	No	No
22	No	B	A	No	No
23	No	B	B	No	Yes
24	Yes	B	B	No	Yes
25	No	B	A	No	Yes
26	No	B	A	No	No
27	No	B	B	No	No
28	Yes	B	B	No	Yes
29	No	B	B	No	No
30	No	B	B	No	Yes
34	No	B	A	No	No
35	No	B	A	No	No
36	No	B	B	No	No
37	No	B	A	No	No
38	No	B	A	No	No
39	No	C	B	No	No
40	No	C	A	No	No
41	No	C	B	No	No
42	No	C	A	No	No
43	No	C	A	No	Yes
44	Yes	C	A	Yes	No
45	Yes	C	B	No	No
46	No	C	A	No	No
47	Yes	C	A	No	No
51	No	C	B	No	No
52	No	C	A	Yes	Yes
53	No	C	B	No	No

Table S7. The metadata of the deceased.

Case no.	drug use group	Batch	Doctor collecting tissue	Alcohol intake	Postmortem interval >6 hours
54	No	C	A	Yes	No
55	No	C	B	Yes	No
56	No	C	A	No	No
57	Yes	C	A	No	No
58	Yes	C	B	No	No
59	No	C	A	Yes	No
60	No	C	A	No	No
